# Onsite Seismic Monitoring Behavior of Undamaged Dams During the 2023 Kahramanmaraş Earthquakes (M7.7 and M7.6)

**DOI:** 10.3390/s24216856

**Published:** 2024-10-25

**Authors:** Alemdar Bayraktar, Mehmet Akköse, Carlos E. Ventura, Tony Y. Yang, Emin Hökelekli

**Affiliations:** 1 Civil Engineering Department, Karadeniz Technical University, Trabzon 61080, Türkiye; 2 Civil Engineering Department, University of British Columbia, Vancouver, BC V6T 1Z4, Canada; ventura@civil.ubc.ca (C.E.V.);; 3 Civil Engineering Department, Bartın University, Bartın 74100, Türkiye

**Keywords:** earthfill dam, concrete-faced rockfill dam, roller-compacted concrete dam, monitored seismic behavior, seismic monitoring, 2023 Kahramanmaraş earthquakes

## Abstract

On 6 February 2023, two major earthquakes struck Türkiye, with their epicenters in the Pazarcık (M7.7; focal depth: 8.6 km) and Elbistan (M7.6; focal depth: 7 km) districts of Kahramanmaraş city. Most of the dams in the earthquake region remained structurally safe and stable. However, 17 dams in Türkiye and 1 dam in Syria were damaged during the 2023 Kahramanmaraş earthquakes. The main objective of this study was to better understand the real seismic behaviors of the dams during the two mainshocks and significant aftershocks. An earthfill dam, a concrete-faced rockfill dam (CFRD), and a roller-compacted concrete (RCC) dam constructed in the disaster area were selected to identify the real seismic behaviors of different types of dams during strong earthquakes. Acceleration records measured at the crest, right and left abutments, and foundations of the selected dams during the 2023 Kahramanmaraş earthquakes were taken into account to determine the real seismic behavior of the dams before, during, and after the earthquakes. The results of this investigation provide valuable insights into the real seismic behaviors of different types of dams in the vicinity of fault lines during strong earthquakes.

## 1. Introduction

Dams are typically designed, constructed, operated, maintained, and monitored by skilled engineers, making them generally resistant to earthquake effects. However, strong earthquakes can still cause damage to dams [1,2,3,4,5,6,7,8,9,10,11,12,13,14,15,16]. The extent of damage to dams during earthquakes is influenced by several factors, including their typology, geometry, construction materials, foundation stratigraphy, and unique design features, as well as the presence of a reservoir and the dam’s past loading history. Additionally, earthquake characteristics—such as magnitude, epicentral distance, depth, and proximity to fault lines—can significantly affect the level of damage [12,13,14,17,18].

Despite advancements in seismic analysis of dams, accurately predicting their behavior during strong ground shaking remains challenging. This is primarily due to the difficulties in modeling the inelastic response of dams, limited information on the spatial variability of ground motion, and other contributing factors. However, seismic instrumentation and monitoring systems allow for real-time observation of dam behavior and structural safety before, during, and after earthquakes. These systems help identify critical dynamic properties of dams, such as internal damping, amplification of ground motion from the foundation or abutments to the crest, wave propagation within the structure, differential movement between abutments, and natural frequencies along with mode shapes.

Several studies have been conducted on the behavior of dams during earthquakes. Seed et al. [10], Tani [11,12,13], and Tani and Nakashima [14] investigated the behavior of large fill dams during seismic events. Pells and Fell [19] reported on the damages and cracking observed in embankment dams due to earthquakes. Matsumoto [20] and Matsumoto et al. [21] assessed the impact of seismic activity on dam safety in Japan. Zhang [22] evaluated the behavior of dams and hydropower structures during the Wenchuan earthquake, which occurred on 12 May 2008, with a magnitude of 8.0 and a focal depth of 15 km in the Wenchuan-Beichuan Longmenshan Fault belt in Sichuan Province. Researchers also analyzed the seismic damage and behavior of earth and concrete-faced rockfill dams during this earthquake [3,23]. Additionally, USCOLD [15] prepared a report on the observed behavior of dams during earthquakes, and Gordan et al. [6] reviewed the dynamic behavior of earth dams and embankments during seismic events.

On 6 February 2023, two powerful earthquakes struck Türkiye, with epicenters in the Pazarcık (M7.7, focal depth: 8.6 km) and Elbistan (M7.6, focal depth: 7 km) districts of Kahramanmaraş, occurring at 04:17 and 13:24 local time, respectively [24]. Later, on 20 February 2023, another earthquake with a magnitude of M6.4 hit Yayladağ, Hatay, at 20:04 local time. These seismic events caused extensive damage to buildings across 11 provinces and severely impacted engineering structures, including dams, bridges, tunnels, and more [2,24,25,26,27,28,29,30,31].

This paper aims to assess the actual seismic behavior of dams located in the earthquake-affected region during the two main shocks and significant aftershocks of the 6 February 2023 Kahramanmaraş earthquakes in Türkiye. The study begins with an overview of the region’s major active faults, seismic activity in Türkiye, and the specific characteristics of the 6 February 2023 earthquakes. It also provides details on the damaged dams, which include zoned earthfill dams, earth core rockfill dams, earth core sand gravel dams, earthfill dams, and concrete-faced rockfill dams. Subsequently, the seismic behavior of an earthfill dam, a concrete-faced rockfill dam (CFRD), and a roller-compacted concrete (RCC) dam in the earthquake region is evaluated separately, examining their behavior before, during, and after the 2023 Kahramanmaraş earthquakes.

## 2. 6 February 2023 Kahramanmaraş, Türkiye, Earthquakes

**Figure 1:**
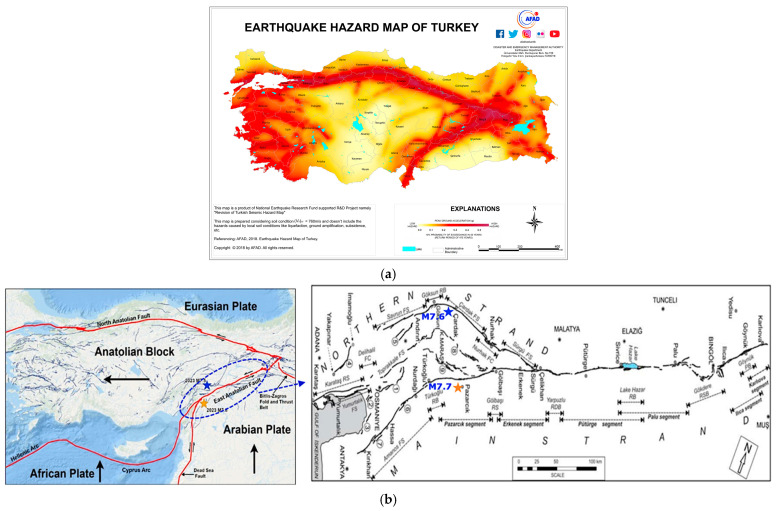
The earthquake hazard map (**a**), and main active faults of Türkiye and the East Anatolian Fault (EAF) system (**b**) [31,35,36].

Türkiye is located in one of the most seismically active regions in the world. The major active faults and the earthquake hazard map of Türkiye published in 2018 are shown in Figure 1. It can be seen from Figure 1 that Türkiye lies on the Anatolian plate and is surrounded by the Arabian, the African, and the Eurasian plates. The tectonic deformation of Türkiye is largely accommodated by two major active faults, including the East Anatolian Fault (EAF) and the North Anatolian Fault (NAF). The fault mechanism of the EAF zone, which is about 450 km long, is the NE-trending left-lateral strike-slip fault system that lies between Karlıova and Hatay [32,33]. The EAF zone extends to the left-lateral strike-slip Dead Sea Fault Zone in the south [34].

On 6 February 2023, at 04:17 a.m. local time (01:17 GMT), with a moment magnitude of M7.7 (Mercalli intensity scale: XI), the Pazarcık (Kahramanmaraş) earthquake occurred on the EAF zone. The epicenter of the Pazarcık (Kahramanmaraş) earthquake was located at 37.288° N, 37.043° E and approximately 40 km north-west of Gaziantep, and 33 km south-east of Kahramanmaraş, with a focal depth of 8.6 km [24]. Approximately 10 min after the mainshock on 6 February 2023, at 04:28 a.m. local time (01:28 GMT), with a moment magnitude of M6.6, a strong aftershock at Nurdağı (Gaziantep) occurred on the EAF zone. The epicenter of the aftershock was located at 37.304° N, 36.92° E and approximately 82 km south-west of Gaziantep city, and 51 km south-west of Kahramanmaraş city, with a focal depth of 6.2 km. Following the first two events, approximately 9 h later, at 13:24 p.m. local time (10:24 GMT), an M7.6 earthquake at Elbistan (Kahramanmaraş) shook the region again. The epicenter of the second mainshock was located at 38.089°N, 37.239° E, approximately 98 km north-west of Adıyaman city, and 62 km north-east of Kahramanmaraş city, with a focal depth of 7.0 km. The Elbistan (Kahramanmaraş) earthquake (M7.6; Mercalli intensity scale: X) occurred along the Çardak-Sürgü fault segment, which is a part of the EAF zone [24]. In addition, on 20 February 2023 at 20:04 local time (17:04 GMT), a large M6.4 earthquake struck Yayladağ, Hatay, within the East Anatolian Fault (EAF) zone, with coordinates N36.037°, E36.021° and a focal depth of 21.73 km [24].

**Figure 2:**
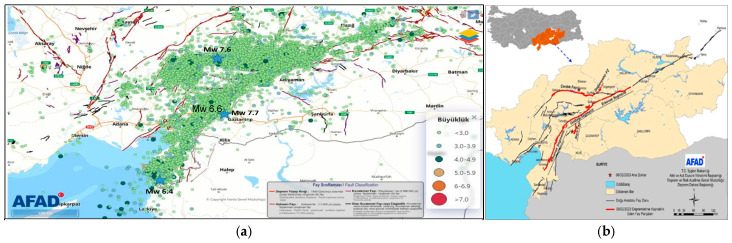
The epicenters of the mainshocks and aftershocks of the February 2023 Kahramanmaraş and Hatay earthquakes (**a**), and the affected region (**b**) [24].

Figure 2 displays the locations of the epicenters of the mainshocks and aftershocks, and the fault lines and the affected area. A total number of 38,000 aftershocks were recorded in the region between February 6 and 6 May 2023 within a 200 km epicenter distance. In total, 579 of these aftershocks had magnitudes exceeding M4.0 [1]. These earthquakes, all of which are unprecedented in recent history in terms of magnitude and coverage, caused major devastation in a total of 11 provinces. The total population of the 11 provinces affected by the earthquake was registered as 14,013,196 persons in 2022 and the total number of buildings in the region was close to 2.6 million [30].

**Figure 3:**
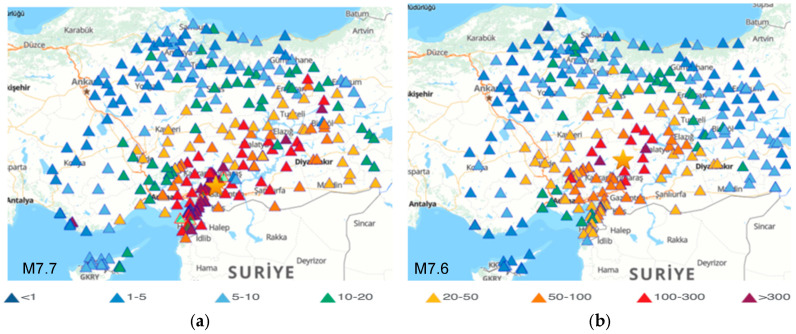
The peak ground accelerations (units in cm/s^2^) of 6 February 2023 Kahramanmaraş, Türkiye, earthquakes: M7.7 (**a**) and M7.6 (**b**) [35].

The strong ground motion data of the 6 February 2023 Kahramanmaraş earthquakes were recorded by the Turkish Accelerometric Database and Analysis System (TADAS) (https://tadas.afad.gov.tr, accessed on 1 August 2024), which is operated by AFAD (Disaster and Emergency Management Presidency) by using more than 800 strong motion stations in Türkiye and Cyprus [35]. The peak ground acceleration (PGA) distributions, indicated by the triangles, recorded at the stations for M7.7 and M7.6 are shown in Figure 3. The peak ground acceleration (PGA), peak ground velocity (PGV) and peak ground displacement (PGD) of the first mainshock (M7.7) were reported as 1372.07 cm/s^2^ (E-W) (TK3135, Repi = 142.15 km), 186.78 cm/s (N-S) (TK3123, Repi = 143.00 km) and 661.90 cm (U-D) (TK0720, Repi = 428.19 km). The PGA, PGV and PGD of the second mainshock (M7.6) were reported as 635.45 cm/s^2^ (N-S) (TK4612, Repi= 66.68 km), 170.79 cm/s (N-S) (TK4612 Repi = 66.68 km) and 614.52 cm (U-D) (TK0720, Repi = 472.58 km) [35].

## 3. Seismic Behaviors of Dams During the 2023 Kahramanmaras Earthquakes

Türkiye presents a challenging tectonic and geological environment for the construction of safe dams. Many dams in Türkiye are located in close proximity to active faults, and some have been built directly across them. There are 140 dams used for irrigation, drinking water, flood control, and electricity generation in the region near the epicenter and fault ruptures of the 2023 Kahramanmaraş earthquakes. This region contains 19% of the total number of dams and 39% of Türkiye’s installed hydraulic capacity [30].

Despite such a high acceleration, most of the dams remained in a structurally safe and stable condition in the earthquake-affected region. Seventeen dams in Türkiye and one dam in Syria were damaged during the 6 February 2023 Kahramanmaraş earthquakes [30]. The estimated damage cost for the seventeen dams was USD 141.8 million [30]. The characteristics, locations and damage levels of the damaged dams are presented in Table 1. The types of damaged dams include zoned earthfill, earth core rockfill, earth core sand gravel, earthfill and concrete-faced rockfill dams. Although the dams in the earthquake-affected region were exposed to two major earthquakes and many aftershocks, the dams experienced minor to major cracks. Nevertheless, they did not fail to retain water and resulted in no uncontrolled release of water from the reservoir.

The horizontal acceleration values at the crest levels of the damaged dams were approximately calculated to be four times greater than at the dam foundation [2]. Longitudinal cracks were observed at the crests and upstream faces of the dams, with their length and depth increasing significantly through the dam crest. The width of the longitudinal cracks at the dam crest was also considerably large. In addition to dam damages in Türkiye, the Medanki dam in Afrin (Syria) was damaged due to the 2023 Kahramanmaraş earthquakes. It is an earthfill dam, and its high and crest length are 75 m and 980 m, respectively. It is located near the south-east border of Türkiye. Cracks occurred along the dam crest; however, there was no water leakage after the earthquakes [2].

The monitoring data for the damaged dams shown in Table 1 were not recorded by AFAD [35] during the February 2023 Kahramanmaraş earthquakes. However, monitoring data were obtained from the undamaged dams in the earthquake-affected region during the February 2023 Kahramanmaraş earthquakes. An earthfill dam, a concrete-faced rockfill dam (CFRD), and a roller-compacted concrete (RCC) dam constructed in the earthquake-prone region were selected to assess the real seismic behavior of different types of dams during strong earthquakes in this study. The real seismic behaviors of these three selected dams, which are the Tahtaköprü Dam, Kavşak Bendi Dam and Feke II Dam, are individually evaluated and discussed in detail below. SARA ACEBOX accelerometers were installed at the aforementioned dams to record the necessary data after the completion of construction [35]. The collected data, sampled at 100 Hz, underwent filtering and baseline correction procedures. A Butterworth filter was applied with low and high cut-off frequencies set at 0.025 Hz and 40.0 Hz, respectively.

### 3.1. Behavior of Earthfill Dams

The Tahtaköprü Dam was selected to evaluate the monitored seismic behavior of earthfill dams during the 6 February 2023 Kahramanmaraş earthquakes. The dam is located approximately 15 km northeast of Hassa District in Hatay Province, near the Syrian border. It was constructed on the Karasu Stream between 1967 and 1975 for irrigation purposes for the type of an earthfill dam. Elevation works were conducted on the dam body between 2017 and 2019, resulting in an increase in the crest level from 407.50 m to 416.50 m [36]. The Tahtaköprü Dam has a height of 55.5 m from the foundation, a crest length of 401.03 m, and a crest width of 10 m. According to the Türkiye Earthquake Hazard Map published in 2018 [35], the maximum ground motion acceleration (PGA) values at Tahtaköprü Dam location for the 475- and 2475-year return periods are 0.465 g and 0.858 g, respectively. According to ICOLD, the 475-year return period corresponds to the Safety Evaluation Earthquake (SEE), while the 2475-year return period corresponds to the Maximum Credible Earthquake (MEC). The dam was designed to withstand 0.25 g [37].

**Figure 4:**
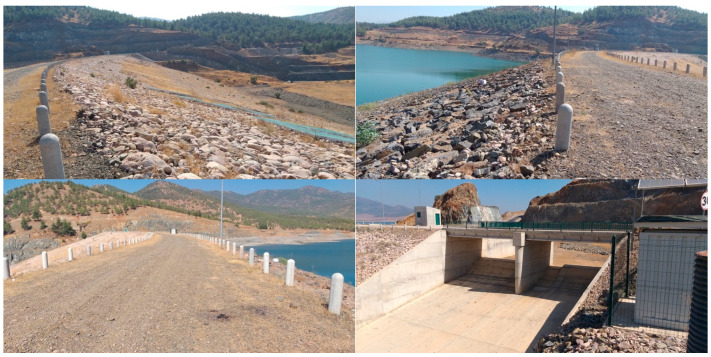
Views from Tahtaköprü dam after the 2023 Kahramanmaraş earthquakes.

**Figure 5:**
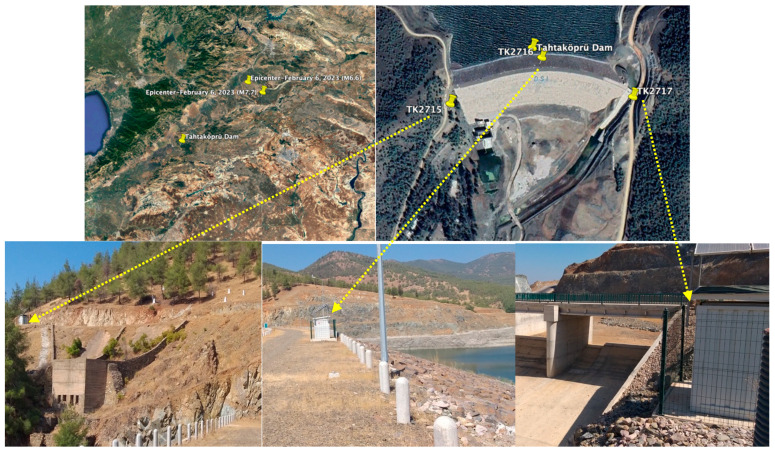
The locations of the main (M7.7) and aftershock (M6.6) epicenters and the Tahtaköprü Dam accelerometer stations.

Some views from the crest, upstream and downstream faces, and spillway of the Tahtaköprü dam after the 2023 Kahramanmaraş earthquakes are shown in Figure 4. The strong ground motion accelerometer stations with three components were installed at the dam crest (TK2716), right abutment (TK2715) and left abutment (TK2717) of the dam on 4 April 2021. Figure 5 displays the epicenters of the 2023 Kahramanmaraş earthquakes and the accelerometer station locations. The left (TK2715) and right (TK2717) abutment stations were located on the bedrock. Elevations of TK2715, TK2716 and TK2717 stations were 431.0 m, 415.0 m and 419.0 m, respectively [35].

**Figure 6:**
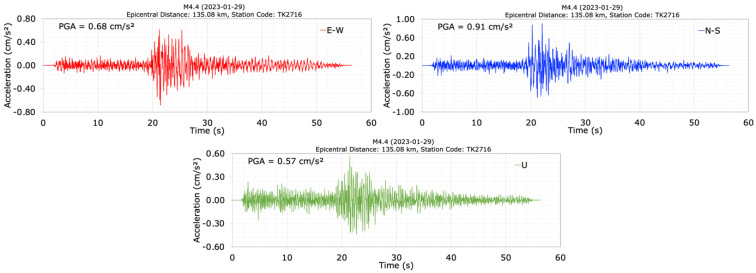
Acceleration time histories at the dam crest station (TK2716) during the M4.4 earthquake.

The acceleration data recorded on the Tahtaköprü dam crest and its abutments were obtained for the M4.4, M7.7, and M6.6 earthquakes. On 27 January 2023, at 16:12, an earthquake with a magnitude of M4.4 occurred, which preceded the M7.7 and M6.6 earthquakes on 6 February 2023. The earthquake (M4.4) had a depth of 18.35 km and an epicenter distance of 135.08 km from the dam. During the M4.4 earthquake, acceleration graphs recorded in the E-W, N-S, and U (vertical) directions at the crest station (TK2716) are plotted in Figure 6. For this earthquake, the maximum accelerations measured in the crest in the E-W, N-S, and U (vertical) directions are 0.68 cm/s^2^, 0.91 cm/s^2^, and 0.57 cm/s^2^, respectively. When evaluating the acceleration records of the M4.4 earthquake in Figure 6, the dominant periods of the dam before the February 2023 Kahramanmaraş earthquakes were calculated as 0.59 s, 0.62 s, and 0.37 s for the E-W (in the dam axis or cross-valley), N-S (in the stream or upstream/downstream direction), and U (vertical) components, respectively. The dominant period corresponds to the largest amplitude value in the Fourier transform of the crest acceleration data.

**Figure 7:**
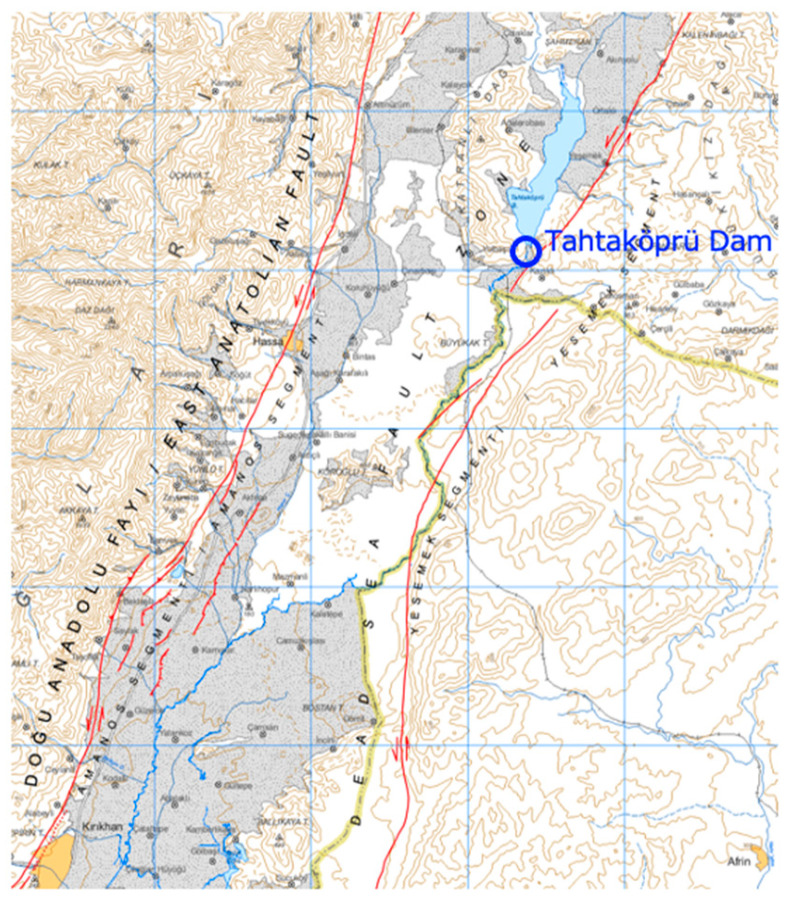
The location of Tahtaköprü Dam as well as the EAF and DSF faults [33].

Acceleration measurements during the 6 February 2023 Kahramanmaraş earthquakes were taken at three locations, the dam crest (TK2716), the right abutment (TK2715), and the left abutment (TK2717), for both the mainshock (M7.7) and the aftershock (M6.6) earthquakes. The M6.6 aftershock occurred approximately 10 min after the M7.7 mainshock. The distances of stations TK2715, TK2716, and TK2717 from the epicenter of the M7.7 mainshock earthquake are 57.62 km, 57.38 km, and 57.34 km, respectively, while the distances from the epicenter of the M6.6 aftershock earthquake are 54.05 km, 53.84 km, and 53.85 km [35]. The epicenters of both earthquakes are very close to each other. Figure 7 shows the locations of the dam and the active faults, named as the East Anatolian Fault (EAF) and the Dead Sea Fault (DSF). The EAF and DSF faults are located within approximately 13 km and 1 km from the dam site, respectively. The 6 February 2023 Kahramanmaraş earthquakes occurred along the East Anatolian Fault (EAF).

**Figure 8:**
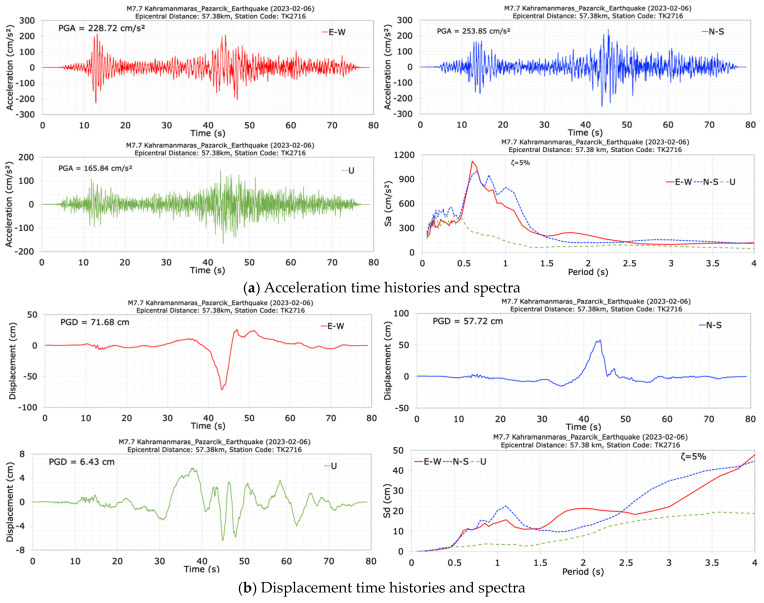
Acceleration (**a**) and displacement (**b**) time histories and spectra at the crest station (TK2716) during the M7.7 earthquake.

Figure 8 displays the time histories and spectra of the accelerations and displacements in the dam axis (E-W), stream (N-S), and vertical (U) directions during the mainshock (M7.7) at the crest station (TK2716). The duration of the records is approximately 80 s. It can be seen from Figure 8 that the seismometer at the crest station (TK2716) recorded peak accelerations of 228.72 cm/s^2^ in the dam axis (E-W), 253.85 cm/s^2^ in the stream (N-S), and 165.84 cm/s^2^ in the vertical (U) directions. The largest crest acceleration occurred in the stream (N-S) direction. Furthermore, Figure 8 demonstrates that the acceleration response spectra values at the crest station (TK2716), with a 5% damping ratio, peak in the period range of 0.5 s to 1.5 s. Two significant pulses of the M7.7 strong earthquake motion can also be observed in all the acceleration time histories recorded at the crest (Figure 8). After the M7.7 mainshock, the dominant periods of the dam in the dam axis (E-W), stream (N-S) and vertical (U) directions were calculated as 0.61 s, 0.83 s and 0.49 s, respectively. The damping ratios for the chosen dams were determined using the Logarithmic Decrement Method, taking into account the maximum acceleration components measured at the dam crest. For the Tahtaköprü earthfill dam, the damping ratios were calculated as 6.70% for the M7.7 earthquake’s N-S component and 10.69% for the M6.6 aftershock’s E-W component, based on PGA data.

The peak displacements at the dam crest in the dam axis (E-W), stream (N-S) and vertical (U) directions are 71.68 cm, 57.72 cm and 6.43 cm, respectively, for the M7.7 earthquake. Although significant horizontal crest displacements were obtained from the accelerations recorded at the crest during the main earthquake (M7.7), during the observational inspections, no permanent deformations or cracks were found in the dam crest, body or foundation, and no water leakage was observed downstream (see Figure 4). It can be stated that the absence of permanent deformations in embankment dams, despite large horizontal displacements during the earthquake, can be attributed to the elastic behavior of materials, effective damping mechanisms, temporary nature of seismic forces, compaction and redistribution of materials, and the dam’s geometric configuration. The effects of these factors can be explained as follows. (1) Elastic behavior of materials: The materials used in embankment dams, such as compacted soil and rock fill, exhibit significant elastic behavior under dynamic loading. During an earthquake, the dam materials can deform elastically, absorbing and then releasing the seismic energy without undergoing permanent deformation. This elastic response is typically reversible, meaning that once the seismic forces subside, the materials return to their original state. (2) Damping mechanisms: Embankment dams have inherent damping mechanisms that dissipate the seismic energy. These mechanisms include internal friction within the dam materials, energy absorption by the foundation, and interaction with the surrounding environment. Effective damping reduces the impact of transient seismic forces, helping to prevent permanent displacements. (3) Temporary deformations: The recorded horizontal displacements during an earthquake are often temporary and can be largely attributed to the inertial forces acting on the dam. These forces cause the dam to move back and forth, resulting in significant but temporary displacements. Once the earthquake ends, the inertial forces cease, allowing the dam to settle back to its original position. (4) Compaction and redistribution of materials: During seismic shaking, the granular materials in the dam body can undergo compaction and slight rearrangement. This process can improve the overall stability and density of the dam, reducing the likelihood of permanent deformations. The materials may become more tightly packed, increasing their resistance to future seismic events. (5) Geometric configuration: The design and geometric configuration of embankment dams play a crucial role in their seismic behavior. Properly designed slopes, adequate freeboard, and well-compacted materials enhance the dam’s ability to withstand large displacements without experiencing permanent damage. These factors collectively ensure that the dam can absorb and dissipate seismic energy without sustaining long-term damage, maintaining its structural integrity and safety.

**Figure 9:**
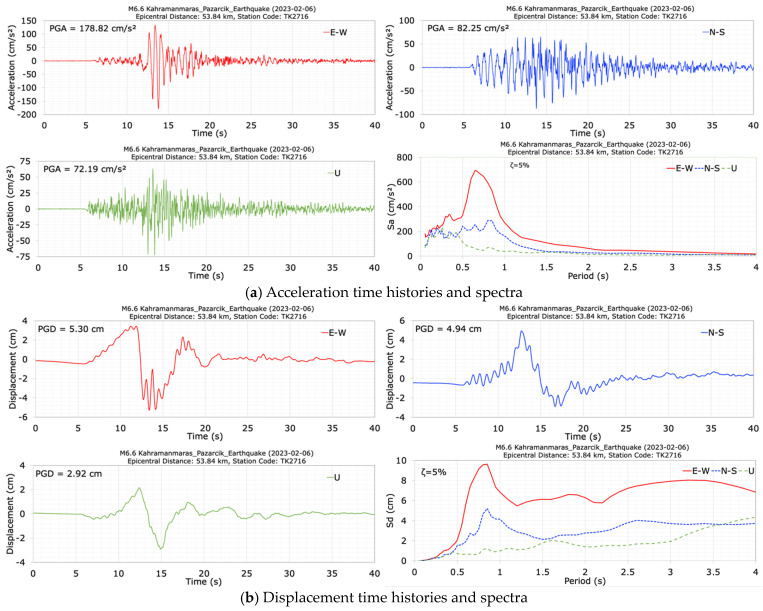
Acceleration (**a**) and displacement (**b**) times histories and spectra at the crest station (TK2716) during the M6.6 earthquake.

After the main shock earthquake (M7.7), an aftershock earthquake with a magnitude of M6.6 and almost the same epicenter distance occurred approximately 10 min later. The acceleration and displacement time histories and spectra at the crest station (TK2716) during the aftershock (M6.6) earthquake in the dam axis (E-W), stream (N-S), and vertical (U) directions are presented in Figure 9. The total duration of the records is approximately 40 s. It can be seen from Figure 9 that the seismometer at the crest station (TK2716) recorded peak accelerations of 178.82 cm/s^2^ in the dam axis direction (E-W), 82.25 cm/s^2^ in the stream direction (N-S), and 72.19 cm/s^2^ in the vertical direction (U). The largest crest acceleration occurred in the dam axis (E-W) direction. The values of horizontal acceleration response spectra calculated for a 5% damping ratio at the crest station have effective values in the period range of 0.5 s to 1.0 s. The obtained peak displacements at the crest point in the E-W, N-S and U directions are 5.30 cm, 4.94 cm and 2.92 cm, respectively. Following the M6.6 aftershock earthquake, the dominant periods of the dam were calculated as 0.73 s in the dam axis (E-W), 0.76 s in the stream (N-S), and 0.41 s in the vertical (U) directions, respectively.

**Figure 10:**
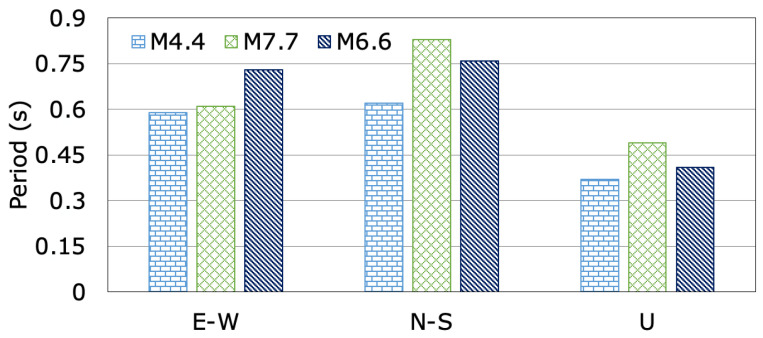
The changes in dominant periods of Tahtaköprü Dam during the M4.4, M7.7, and M6.6 earthquakes.

Figure 10 displays the variations in the dominant periods of the dam in the dam axis (E-W), stream (N-S), and vertical (U) directions for the M4.4, M7.7, and M6.6 earthquakes. It can be seen from Figure 10 that the dominant periods of the dam in the dam axis (E-W), stream (N-S) and vertical (U) directions show a noticeable change before, during, and after the mainshock (M7.7). The dominant periods of the dam following the mainshock (M7.7) and aftershock (M6.6) were found to be longer compared to the initial earthquake (M4.4) that occurred before the February 2023 earthquakes. The recorded dominant periods of the Tahtaköprü earthfill dam, with a crest length-to-height ratio of 7.23, during the M4.4, M7.7, and M6.6 earthquakes in the dam axis (E-W), stream (N-S), and vertical (U) directions range from 0.59 s to 0.61 s, 0.62 s to 0.83 s, and 0.37 s to 0.41 s, respectively. The increases in the dam-dominant period in the stream direction (N-S) for the M7.7 and M6.6 earthquakes are 25.30% and 18.42%, respectively. The dominant periods in the dam axis (E-W) direction increased by 3.28% and 19.18% for the main (M7.7) and aftershock (M6.6), respectively, while those in the vertical (U) direction increased by 24.49% and 9.76%, respectively. The dam exhibited changes in its dominant periods when subjected to the pre-shock, main shock, and aftershock, but no damage was observed. This situation can be attributed to the elastic behavior of the materials, the effective damping mechanisms, the temporary nature of the seismic forces, changes in the frequency content of the of the input ground motions, the compaction and redistribution of the materials, and the geometric configuration of the dam.

**Figure 11:**
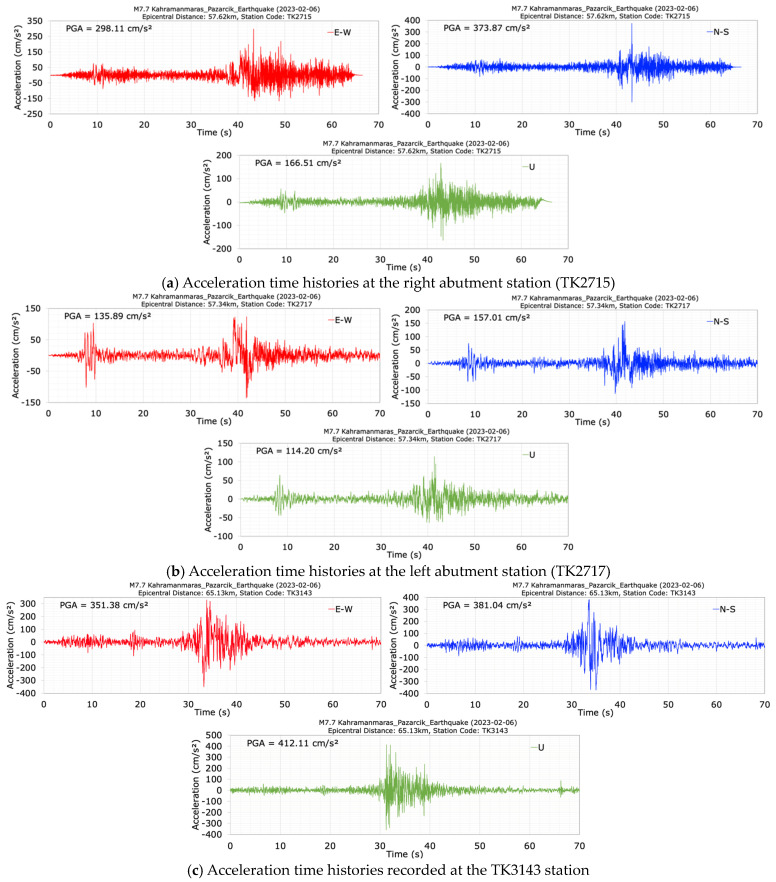
Acceleration time histories recorded at the right abutment station (TK2715) (**a**), the left abutment station (TK2717) (**b**), and at the TK3143 station during the mainshock (M7.7).

The acceleration records measured during the M7.7 mainshock earthquake on the right (TK2715) and left (TK2717) abutments of the dam are depicted in Figure 11. The maximum accelerations in the dam axis (E-W), stream (N-S), and vertical (U) directions recorded at the crest (TK2716), the left (TK2717) and right (TK2715) abutments, the magnification ratios of the acceleration components relative to each other, and the acceleration magnifications at the crest relative to the right and left abutments are summarized in Table 2. A peak horizontal acceleration of 373.87 cm/s^2^ was recorded in the stream (N-S) direction on bedrock at the right abutment (TK2715) that is 16.0 m above the dam crest, while a peak horizontal acceleration of 157.01 cm/s^2^ was measured in the stream (N-S) direction on bedrock at the left abutment (TK2717) that is 4.0 m above the dam crest. The maximum acceleration ratios between the N-S and E-W components (N-S/E-W) recorded at the crest, left and right abutments were calculated as 1.11, 1.16, and 1.25, respectively. The maximum N-S/E-W increase ratio obtained at the right abutment was 1.25. The maximum U/E-W and U/N-S acceleration ratios occurred at the left abutment, with values of 0.84 and 0.73, respectively.

The maximum increase ratios (Crest/LA) from the left abutment (LA) to the crest are 1.62, 1.68 and 1.45 in the stream direction (N-S), dam axis (E-W) and vertical (U) directions, respectively. The ratios (Crest/RA) from the right abutment (RA) to the crest in the same direction are 0.68, 0.77 and 0.99, respectively. The crest acceleration ratios at the left abutment are larger than the right abutment. The maximum amplification at the dam crest occurred in the dam axis (E-W) direction, with a value of 1.68. For the M7.7 earthquake, an increase in crest accelerations can be observed in comparison to the accelerations on the left abutment, while a decrease in crest accelerations can be observed in comparison to the accelerations on the right abutment. The ratios between left (LA) and right (RA) abutment accelerations (LA/RA) in the stream (N-S), dam axis (E-W), and vertical (U) directions are 0.42, 0.46, and 0.69, respectively. For the M7.7 earthquake, the accelerations recorded at the right abutment of the dam are greater than those recorded at the left abutment.

The acceleration data recorded at the TK3143 station, located 11.9 km west of the Tahtaköprü dam, during the first M7.7 mainshock are shown in Figure 11c. This station, with an epicentral distance of 65.13 km, is located on moderately stiff ground with a shear wave velocity (Vs30) of 444 m/s [35]. The epicenter distances of the right and left abutments of the dam are 57.62 km and 57.32 km, respectively. From Figure 11c, it can be observed that the maximum accelerations recorded at the TK3143 station during the M7.7 earthquake in the E-W, N-S and vertical directions are 351.38 cm/s^2^, 381.04 cm/s^2^ and 412.11 cm/s^2^, respectively. The acceleration values recorded at the left and right abutments of the dam during the same earthquake were 135.89 cm/s^2^, 157.01 cm/s^2^, 114.20 cm/s^2^; 298.11 cm/s^2^, 373.87 cm/s^2^, and 166.51 cm/s^2^ for the E-W, N-S, and vertical components, respectively. The acceleration values at TK3143 are higher compared to those at the dam abutments. Furthermore, the frequency content of the acceleration records at the dam abutments differs from that of the TK3143 station data. This indicates that local ground conditions play a significant role in the intensity and frequency components of seismic waves.

**Figure 12:**
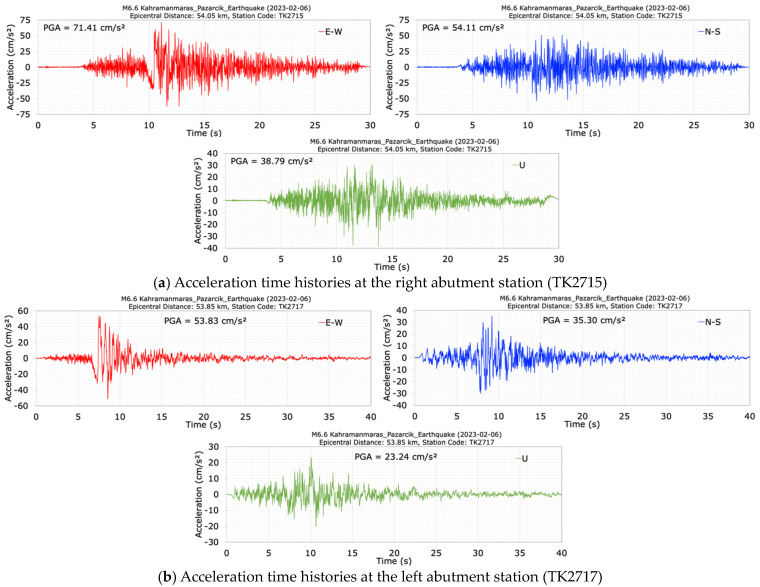
Acceleration time histories recorded at the right (TK2715) (**a**) and the left (TK2717) (**b**) abutment stations during the aftershock (M6.6).

The acceleration records at the right (TK2715) and left (TK 2717) abutments of the dam during the M6.6 aftershock earthquake are plotted in Figure 12. Table 3 summarizes the maximum acceleration ratios measured in the dam axis (E-W), stream (N-S), and vertical (U) directions at the crest (TK2716), left (TK2717), and right (TK2715) abutments for the M6.6 aftershock. During the M6.6 aftershock earthquake, peak accelerations in the E-W, N-S, and vertical (U) directions were recorded at the crest (TK2716) as 178.82 cm/s^2^, 82.25 cm/s^2^, and 72.19 cm/s^2^, respectively. The maximum acceleration ratios between the horizontal components (N-S/E-W) measured at the crest, the left and right abutments are 0.46, 0.66, and 0.76, respectively. The maximum acceleration ratio between the U and E-W components (U/E-W) was 0.54 at the right abutment while the maximum acceleration ratio between the U and N-S components (U/N-S) was calculated as 0.88 at the crest.

The crest acceleration amplification ratios (Crest/LA) relative to those recorded at the left abutment in the E-W, N-S, and U (vertical) directions are 3.32, 2.33, and 3.11, respectively. However, the crest acceleration amplification ratios (Crest/RA) relative to those recorded at the right abutment in the dam axis (E-W), stream (N-S), and vertical (U) directions are 2.50, 1.52, and 1.86, respectively. Significant increases in crest accelerations compared to the left and right abutment accelerations were observed during the M6.6 aftershock earthquake. The maximum crest acceleration amplification ratio during the M6.6 aftershock was calculated as 3.32 in the cross-canyon direction (E-W). The acceleration ratios (LA/RA) between the left abutment and the right abutment in the stream (N-S), dam axis (E-W) and vertical (U) directions were recorded as 0.65, 0.75 and 0.60, respectively. The accelerations recorded at the right abutment of the dam during the M6.6 aftershock also exceed those recorded at the left abutment. It is thought that the reason why the accelerations at the right abutment station, which is 12 m above the left abutment station point, are greater than those at the left abutment station is due to the difference in elevation and soil properties as well as the topographic amplification in the canyon.

Upon comparing Table 2 and Table 3, it becomes evident that the PGAs recorded at the right abutment during the M7.7 mainshock have the highest values, while the PGAs at the crest during the M6.6 aftershock exhibit the largest values. An analysis of Figure 11a reveals spikes in both the E-W and N-S directions at the right abutment during the M7.7 earthquake. If the spikes in the E-W and N-S acceleration records shown in Figure 11a are excluded, the PGA values measured at the right abutment for the E-W and N-S components during the M7.7 earthquake are 218.42 cm/s^2^ and 189.72 cm/s^2^, respectively. Not considering the spikes in the acceleration records at the right abutment during the M7.7 earthquake reveals higher horizontal PGA values recorded at the dam crest when compared to the right abutment, similar to the situation during the M6.6 earthquake.

### 3.2. Behavior of Concrete-Faced Rockfill Dams (CFRDs)

**Figure 13:**
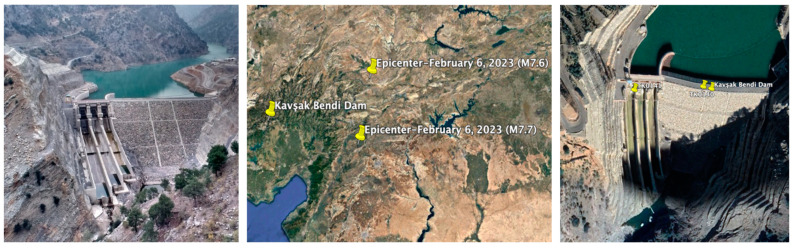
The locations of the first (M7.7) and the second main shock (M7.6) epicenters and the Kavşak Bendi dam accelerometer stations.

The Kavşak Bendi Dam was chosen to assess the real seismic behavior of concrete-faced rockfill dams during the 6 February 2023 earthquakes. This dam is a concrete-faced rockfill dam (CFRD) constructed for the purpose of energy production between the years 2009–2014 on Seyhan River in the Kozan District of Adana Province, Türkiye. The current dam has a height of 88 m from the foundation, 73 m from the thalweg, a crest length of 186 m, a crest width of 8 m, and a crest elevation of 323 m. According to the Türkiye Earthquake Hazard Map published in 2018 [35], the PGA values for the 475 and 2475-year return periods at the location of Kavşak Bendi Dam are calculated as 0.239 g and 0.493 g, respectively. The strong ground motion accelerometers with three components were installed at the dam crest (TK0140) and the right abutment bedrock (TK0141) of the dam on 29 December 2022. Figure 13 shows a view of the dam, the locations of the dam accelerometers, and the epicenters of the first (M7.7) and the second (M7.6) mainshocks of the 6 February 2023 Kahramanmaras earthquakes. Elevations of the TK0140 and TK0141 stations are 322.0 m and 312.0 m, respectively [35].

Prior to the Kahramanmaraş earthquakes on 6 February 2023, no earthquake was recorded by the sensors at the Kavşak Bendi Dam (AFAD, 2023b). Therefore, the dominant period of the dam could not be determined before the 6 February 2023 Kahramanmaraş earthquakes. However, accelerations were recorded at the dam crest (TK0140) and right abutment (TK0141) stations for the first (M7.7) and the second (M7.6) mainshocks of the 6 February 2023 Kahramanmaras earthquakes. TK0140 and TK0141 stations were located at distances of 137.11 km and 137.23 km, respectively, from the epicenter of the first mainshock (M7.7) earthquake, and at distances of 161.17 km and 161.28 km, respectively, from the epicenter of the second mainshock (M7.6) earthquake [35].

**Figure 14:**
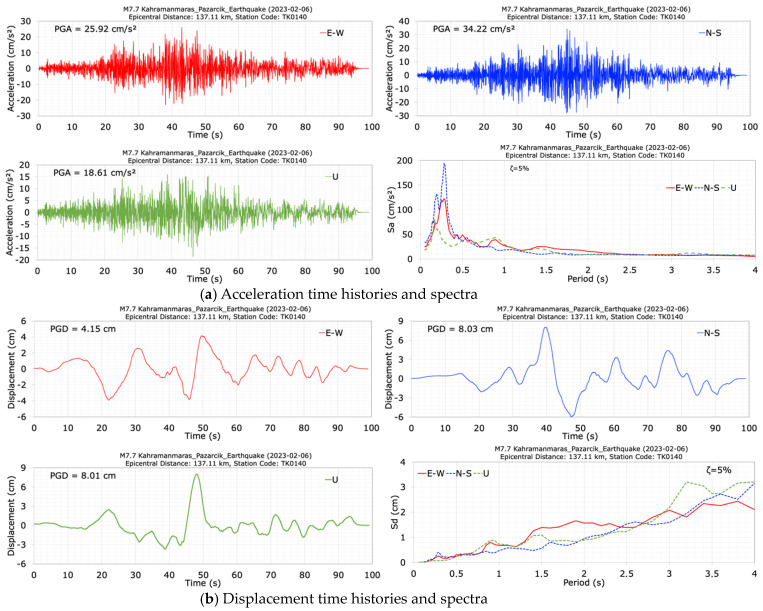
Acceleration (**a**) and displacement (**b**) time histories and spectra at the dam crest station (TK0140) during the M7.7 earthquake.

The acceleration and the displacement time histories and spectra at the dam crest station (TK0140) in the E-W (along dam axis), N-S (along the stream) and U (vertical) directions of the mainshock (M7.7) are shown in Figure 14. The duration of the acceleration records is approximately 100 s. It can be seen from Figure 14 that the seismometer at the dam crest station (TK0140) recorded peak accelerations of 25.92 cm/s^2^ in the dam axis (E-W), 34.22 cm/s^2^ in the stream (N-S), and 18.61 cm/s^2^ in the vertical (U) directions. The largest crest acceleration occurred in the stream (N-S) direction. Additionally, Figure 14 shows that the values of acceleration response spectra calculated for a 5% damping ratio at the dam crest station have high values between the period range of 0.1 s to 0.5 s. The peak displacements at the crest point in the dam axis (E-W), stream (N-S) and vertical (U) directions are 4.15 cm, 8.03 cm and 8.01 cm for the M7.7 earthquake, respectively. Following the first mainshock (M7.7), the dominant periods of the dam in the dam axis (E-W), stream (N-S) and vertical (U) directions were calculated as 0.25 s, 0.27 s, and 0.17 s, respectively.

**Figure 15:**
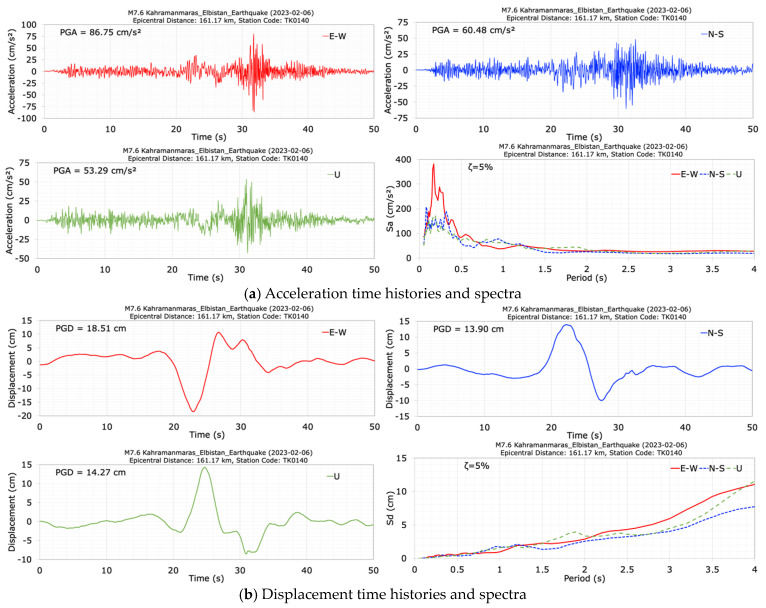
Acceleration (**a**) and displacement (**b**) time histories and spectra at the dam crest station (TK0140) during the M7.6 earthquake.

The acceleration and displacement time histories and spectra at the dam crest station (TK0140) in the E-W, N-S and vertical (U) directions of the second mainshock (M7.6) are shown in Figure 15. The duration of the records is 50 s. The seismometer at the dam crest station (TK0140) measured peak accelerations of 86.75 cm/s^2^ in the dam axis (E-W), 60.48 cm/s^2^ in the stream (N-S), and 53.29 cm/s^2^ in the vertical (U) directions. The largest crest acceleration of 86.75 cm/s^2^ was recorded in the dam axis (E-W) direction. For the N-S component of the M7.7 earthquake and the E-W component of the M7.6 earthquake, the calculated damping ratios at the dam crest of the Kavşak Bendi cocrete-faced rockfill dam are 4.26% and 7.98%, respectively, based on PGA data.

The horizontal acceleration response spectra calculated for a 5% damping ratio show large values between the period range of 0.1 s to 0.5 s. The obtained peak displacements for the crest point in the dam axis (E-W), stream (N-S) and vertical (U) directions are 18.51 cm, 13.90 cm and 14.27 cm, respectively. After the second mainshock earthquake with a magnitude of M7.6, which occurred 9 h after the first mainshock (M7.7), the dominant periods of the dam were calculated as 0.27 s in the dam axis (E-W), 0.30 s in the stream (N-S), and 0.20 s in the vertical (U) directions.

**Figure 16:**
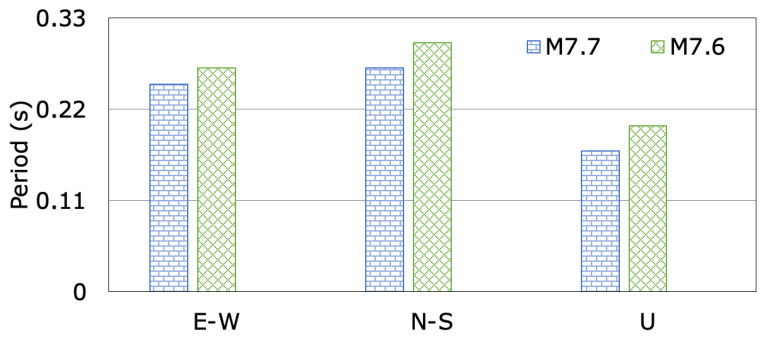
The changes in dominant periods of Kavşak Bendi Dam during the M7.7 and M7.6 earthquakes.

The changes in the dominant periods of the dam, calculated from the analysis of accelerations measured at the crest station (TK0140) following the M7.7 and M7.6 mainshocks, are illustrated in Figure 16 for the dam axis (E-W), stream (N-S), and vertical (U) directions. The recorded dominant periods of the Kavşak Bendi concrete-faced rockfill dam, with a crest length-to-height ratio of 3.67, during the M7.7 and M7.6 earthquakes in the dam axis (E-W), stream (N-S), and vertical (U) directions range from 0.25 s to 0.27 s, 0.27 s to 0.30 s, and 0.17 s to 0.20 s, respectively. The dominant periods of the dam in the stream (N-S), dam axis (E-W) and vertical (U) directions show a slight change during the two mainshocks. The dominant periods of the dam due to the second mainshock (M7.6) lengthened according to the earthquake (M7.7). The increase ratios of the dominant periods in the dam axis (E-W), stream (N-S) and vertical (U) directions during the second mainshock (M7.6) are 7.41%, 10.0% and 15.0%, respectively. The accelerations of the M7.6 earthquake recorded on the dam crest are larger than the accelerations of the M7.7 earthquake. It was observed that the periods of the dam increase with increasing the earthquake acceleration values. The M7.7 and M7.6 earthquakes did not result in any visible seismic damage, crack, or deformation to the Kavşak Bendi dam body or the site (Figure 13).

**Figure 17:**
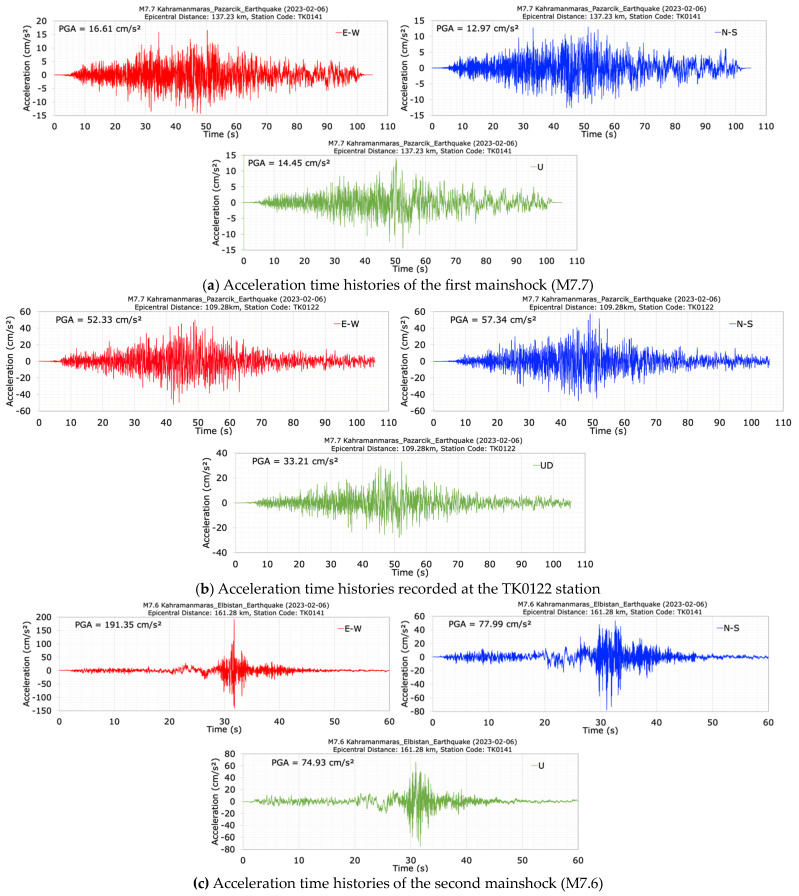
Acceleration time histories at the right abutment station (TK0141) (**a**) and TK0122 station (**b**) during the first (M7.7), and the second (M7.6) (**c**) mainshocks.

The acceleration records measured at the right abutment (TK0141) of the dam during the M7.7 and M7.6 mainshocks are plotted in Figure 17. Table 4 summarizes the maximum accelerations recorded at both the dam crest (TK0140) and the right abutment (TK0141) stations, and the crest amplification ratios relative to the right abutment in the E-W, N-S, and vertical (U) directions. The right abutment station (TK0141) is located 10.0 m below the dam crest station (TK0140). For the M7.7 earthquake, the acceleration amplification ratios at the dam crest relative to the right abutment are calculated as 1.56, 2.64 and 1.29 for the dam axis (E-W), stream (N-S), and vertical (U) directions. However, during the M7.6 earthquake, the acceleration amplification ratios at the dam crest relative to the right abutment for the E-W, N-S, and U components are 0.45, 0.78, and 0.71, respectively. The maximum crest acceleration amplification ratios for the M7.7 and M7.6 earthquakes in the N-S direction are 2.64 and 0.78, respectively. During the M7.7 earthquake, there are significant increases in crest accelerations relative to the right abutment for all acceleration components, whereas the M7.6 earthquake results in significant decreases in crest accelerations relative to the right abutment. This is thought to be due to spikes in the acceleration records of the M7.6 earthquake.

The acceleration data recorded at the TK0122 station, located 29.33 km south-east of the Kavşak Bendi dam, during the M7.7 mainshock are illustrated in Figure 17b. This station, situated on moderately stiff ground with a shear wave velocity (Vs30) of 501 m/s, is at an epicentral distance of 109.28 km. The right abutment of the dam is 137.23 km from the M7.7 earthquake epicenter. As shown in Figure 17b, the maximum accelerations recorded at the TK0122 station during the M7.7 earthquake in the E-W, N-S, and vertical directions are 52.33 cm/s^2^, 57.34 cm/s^2^, and 33.21 cm/s^2^, respectively. In contrast, the recorded acceleration values at the right abutment of the dam during the same event are 16.61 cm/s^2^, 12.97 cm/s^2^, and 14.45 cm/s^2^ for the E-W, N-S, and vertical (U) components, respectively. Although the acceleration values at TK0122 are higher than those at the dam abutment, the frequency content of the acceleration records at the dam abutments is similar to that of the TK0122 station data.

### 3.3. Behavior of Roller-Compacted Concrete Dams (RCCs)

**Figure 18:**
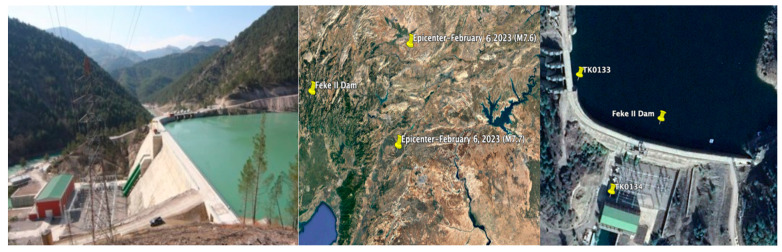
The locations of the first (M7.7) and the second main shock (M7.6) epicenters and the Feke II dam accelerometer stations.

Feke II Dam, located on Göksu River in the Feke District of Adana Province, Türkiye, was constructed between 2009 and 2011 years for the purpose of energy production, using the roller-compacted concrete construction method. The dam’s height from the foundation is 71.0 m, and its crest length is 256.50 m. According to the Türkiye Earthquake Hazard Map published in 2018 [35], the peak ground acceleration (PGA) values of the Feke II Dam location are calculated as 0.254 g and 0.516 g for the 475 and 2475-year return periods, respectively. The strong ground motion accelerometers with the three components were installed at the dam crest (TK0133) and foundation (TK0134) of Feke II Dam on 25 October 2021. Figure 18 provides a view of the dam and the locations of the epicenters of the M7.7 and M7.6 earthquakes, as well as the accelerometer positions. The TK0134 station was situated on the bedrock. The elevation of the dam’s thalweg is 485 m, while the elevations of TK0133 and TK0134 stations are 546 m and 494.0 m, respectively [35].

**Figure 19:**
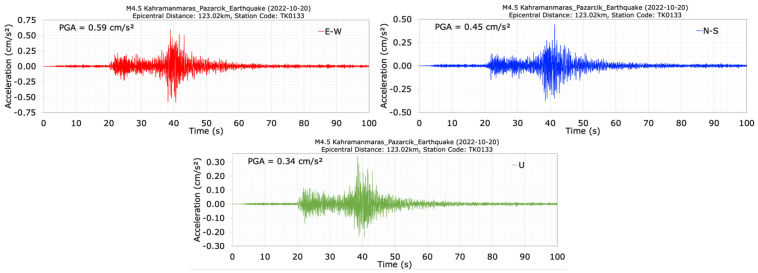
Acceleration time histories at the dam crest station (TK0133) during the M4.5 earthquake.

Prior to the 2023 Kahramanmaraş earthquakes, an earthquake was recorded at the dam on 20 October 2022, at 11:34 am, with a magnitude of M4.5, a depth of 7.64 km, and an epicenter distance of 123.02 km. Acceleration time histories measured in the E-W (dam axis), N-S (stream), and vertical (U) directions at the crest station (TK0133) during the M4.5 earthquake are depicted in Figure 19. The maximum accelerations measured at the dam crest during the M4.5 earthquake are 0.59 cm/s^2^, 0.45 cm/s^2^, and 0.34 cm/s^2^ for the E-W, N-S, and U directions, respectively. When evaluating the acceleration records in Figure 19, the dominant periods of the dam before the Kahramanmaraş earthquakes on 6 February 2023 are calculated as 0.48 s, 0.33 s, and 0.30 s, respectively, for the E-W (dam axis), N-S (stream), and vertical (U) directions.

**Figure 20:**
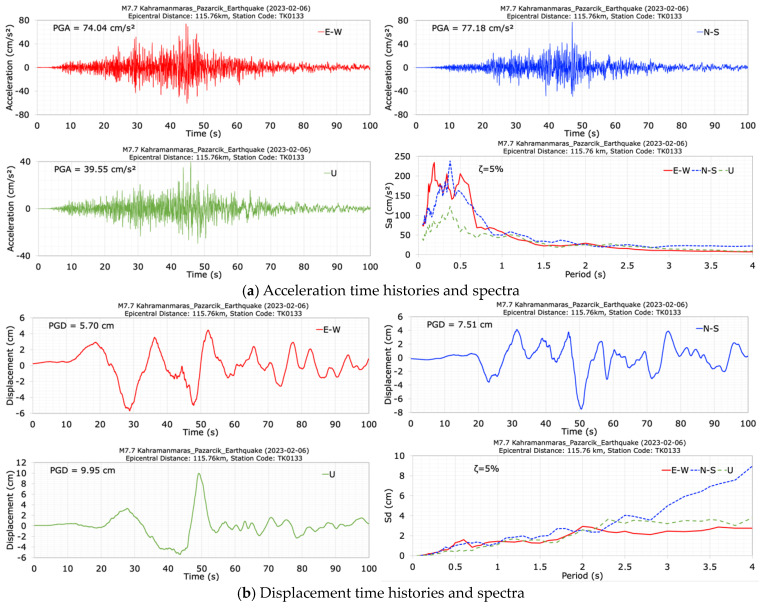
Acceleration (**a**) and displacement (**b**) time histories and spectra at the dam crest station (TK0133) during the M7.7 earthquake.

The accelerations of the 6 February 2023 Kahramanmaras earthquakes were recorded at the dam crest (TK0133) and foundation (TK0134) stations for the first (M7.7) and the second (M7.6) mainshocks. The TK0133 and TK0134 stations are located at distances of 115.76 km and 115.66 km from the epicenter of the first mainshock (M7.7), and at distances of 126.52 km and 126.52 km, respectively, from the epicenter of the second mainshock (M7.6) [35]. The acceleration and displacement time histories and spectra at the crest station (TK0133) in the E-W (dam axis), N-S (stream), and vertical (U) directions are displayed in Figure 20 for the first mainshock (M7.7). The duration of the record is approximately 100 s. It can be seen from Figure 20 that the seismometer at the dam crest station (TK0133) measured peak accelerations of 74.04 cm/s^2^ in the dam axis (E-W), 77.18 cm/s^2^ in the stream (N-S), and 39.55 cm/s^2^ in the vertical (U) directions. The largest crest acceleration occurred in the stream (N-S) direction. Additionally, Figure 20 shows that the values of acceleration response spectra calculated for a 5% damping ratio at the dam crest station (TK0133) have high values between the period range of 0.1 s to 0.75 s. The peak displacements at the crest point in the dam axis (E-W), stream (N-S) and vertical (U) directions are 5.70 cm, 7.51 cm and 9.95 cm for the M7.7 earthquake. Following the first mainshock earthquake of M7.7, the dominant periods of the dam were calculated as 0.41 s in the dam axis (E-W), 0.50 s in the stream (N-S), and 0.38 s in the vertical (U) directions.

**Figure 21:**
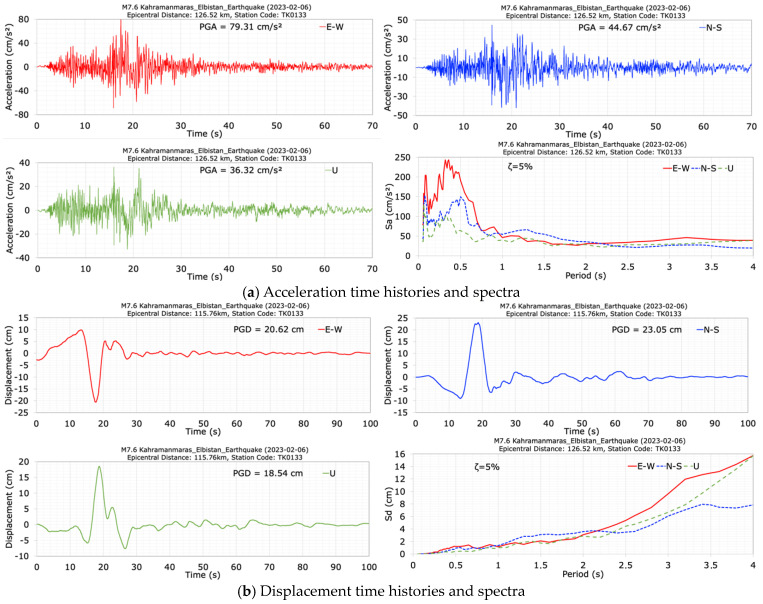
Acceleration (**a**) and displacement (**b**) time histories and spectra at the dam crest station (TK0133) during the M7.6 earthquake.

The acceleration and displacement time histories recorded at the dam crest station (TK0133) in the E-W (along the dam axis), N-S (along the stream) and vertical (U) directions are shown in Figure 21 for the second mainshock earthquake (M7.6). The duration of the records is approximately 70 s. The peak accelerations of 79.31 cm/s^2^ in the dam axis direction (E-W), 44.67 cm/s^2^ in the stream (N-S), and 36.32 cm/s^2^ in the vertical (U) directions were measured at the dam crest station (TK0133). The largest crest acceleration occurred in the dam axis (E-W) direction. The damping ratios calculated for the M7.7 earthquake’s N-S component and the M7.6 earthquake’s E-W component, taking into account the maximum acceleration components recorded at the crest of the Feke II RCC dam, are 3.47% and 7.06%, respectively. The values of horizontal acceleration response spectra calculated for a 5% damping ratio at the dam crest station (TK0133) have effective values between the period range of 0.1 s to 0.75 s. The obtained peak displacements for the crest point in the E-W (dam axis), N-S (stream) and vertical (U) directions are 20.62 cm, 23.05 cm and 18.54 cm, respectively. After the second mainshock (M7.6), which occurred 9 h after the first mainshock (M7.7), the dominant periods of the dam in the dam axis (E-W), stream (N-S), and vertical (U) directions are calculated as 0.40 s, 0.40 s, 0.33 s, respectively.

**Figure 22:**
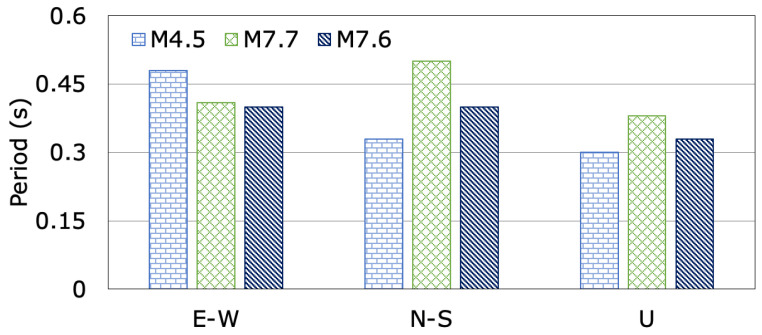
The changes in dominant periods of Feke II Dam during the M4.5, M7.7 and M7.6 earthquakes.

The changes in the dam dominant periods in the dam axis (E-W), stream (N-S), and vertical (U) directions, calculated from the evaluation of acceleration records measured at the dam crest station (TK0133) for the M4.5, M7.7 and M7.6 earthquakes, are illustrated in Figure 22. The recorded dominant periods of the Feke II roller-compacted concrete dam, with a crest length-to-height ratio of 3.61, during the M4.5, M7.7 and M7.6 earthquakes in the dam axis (E-W), stream (N-S), and vertical (U) directions range from 0.48 s to 0.41 s, 0.33 s to 0.40 s, and 0.30 s to 0.33 s, respectively. It can be seen from Figure 22 that the dominant periods of the dam in the dam axis (E-W), stream (N-S), and vertical (U) directions show a noticeable change before, during, and after the mainshocks. During the first (M7.7) and second (M7.6) mainshocks, the dominant periods in the stream (N-S) and vertical (U) directions lengthened according to the earthquake (M4.5). The increases in the dominant periods in the stream direction (N-S) for the M7.7 and M7.6 earthquakes are 34% and 17.5%, respectively. While the dominant periods in the dam axis (E-W) decrease by 17.07% and 20.0%, those in the vertical (U) direction increase by 21.05% and 9.09% after the first (M7.7) and second (M7.6) mainshocks. The first (M7.7) and the second (M7.6) mainshock earthquakes did not cause any visible seismic damage, cracks or deformation to the dam body or the site (Figure 18).

**Figure 23:**
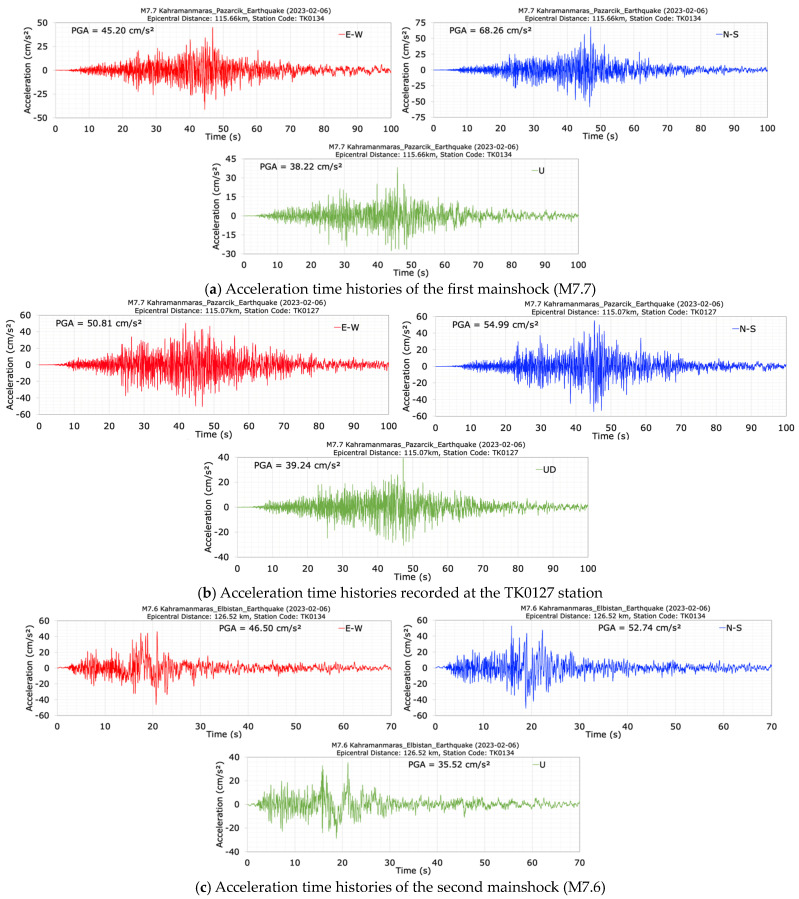
Acceleration time histories at the foundation station (TK0134) (**a**) and TK0127 station (**b**) during the first (M7.7) and the second (M7.6) (**c**) mainshocks.

The acceleration time histories measured at the foundation of the dam (TK0134) during the M7.7 and M7.6 mainshock earthquakes are depicted in Figure 23. Maximum accelerations measured at the dam crest (TK0133) and the foundation (TK0134) stations, and crest amplification ratios relative to the foundation in the E-W, N-S, and vertical (U) directions are summarized in Table 5. The station at the foundation (TK0134) was located 52.0 m below the dam crest station (TK0133). For the M7.7 earthquake, the crest acceleration amplification ratios relative to the foundation in the E-W (dam axis), N-S (stream), and U (vertical) directions were calculated as 1.64, 1.13, and 1.03, respectively. The corresponding acceleration amplification ratios in the E-W, N-S, and vertical directions were 1.71, 0.85, and 1.02 for the M7.6 earthquake. During the M7.7 earthquake, an increase in crest accelerations relative to the foundation was observed for all acceleration components. For the M7.6 earthquake, an increase in crest accelerations relative to the foundation was observed in the E-W (dam axis) and vertical (U) directions, while a decrease was observed in the N-S (stream) direction. The maximum crest acceleration amplification ratios for the M7.7 and M7.6 earthquakes were calculated in the N-S (stream) direction as 1.13 and 0.85, respectively.

The acceleration data recorded at the TK0127 station, located 9.44 km northeast of the Feke II dam during the M7.7 mainshock, is shown in Figure 23b. This station, situated on moderately stiff ground with a shear wave velocity (Vs30) of 583 m/s, is 115.07 km from the earthquake epicenter. The right foundation of the dam is located 115.66 km from the M7.7 epicenter. As seen in Figure 23b, the maximum accelerations recorded at the TK0127 station during the M7.7 earthquake in the E-W, N-S, and vertical directions are 50.81 cm/s^2^, 54.99 cm/s^2^, and 35.52 cm/s^2^, respectively. In comparison, the acceleration values recorded at the right abutment of the dam during the same event are 45.20 cm/s^2^, 68.26 cm/s^2^, and 38.22 cm/s^2^ for the E-W, N-S, and vertical (U) components, respectively. The acceleration values at TK0127 are comparable to those at the dam foundation. Additionally, the frequency content of the acceleration records at the dam foundation is similar to that of the TK0127 station data.

## 4. Conclusions

The seismic behavior of undamaged earthfill, concrete-faced rockfill (CFRD), and roller-compacted concrete (RCC) dams subjected to the 6 February 2023 Kahramanmaraş earthquakes in Türkiye was evaluated and discussed in this paper. This evaluation considers the acceleration data recorded at the dam crests, abutments and foundations during the pre-shock (M4.4), main shocks (M7.7 and M7.6), and aftershock (M6.6). The findings are outlined as follows:

- The M7.7 and M7.6 mainshocks, along with the M6.6 aftershock, did not cause any visible seismic damage, cracks, or permanent deformations to the Tahtaköprü earthfill, Kavşak Bendi concrete-faced rockfill or Feke II roller-compacted concrete dam dams.
- The dominant periods of the Tahtaköprü earthfill, Kavşak Bendi concrete-faced rockfill, and Feke II roller-compacted concrete dams in the stream, dam axis, and vertical directions generally increased by different ratios following the two main shocks (M7.7 and M7.6) and the aftershock (M6.6).
- The maximum damping ratios for the Tahtaköprü earthfill, Kavşak Bendi concrete-faced rockfill, and Feke II RCC dams during the earthquakes were 10.69%, 7.98% and 7.06%, respectively.
- The Tahtaköprü earthfill dam experienced significant horizontal crest displacements during the first main shock (M7.7). However, no permanent deformation was observed at the dam crest or the body. This can be attributed to the elastic behavior of materials, effective damping mechanisms, temporary nature of seismic forces, compaction and redistribution of materials, and the dam’s geometric configuration.
- The acceleration amplification ratios at the crest of the Tahtaköprü earthfill, Kavşak Bendi concrete-faced rockfill and Feke II RCC dams in the stream, dam axis, and vertical directions generally increased in varying amounts relative to the abutments and foundation following the two mainshocks (M7.7 and M7.6) and the aftershock (M6.6).
- The right and left abutments of the Tahtaköprü earthfill, Kavşak Bendi concrete-faced rockfill and Feke II RCC dams exhibited significantly different behaviors during the two mainshocks (M7.7 and M7.6) and the aftershock (M6.6).
- The acceleration time histories recorded at the crests of the dams exhibit distinct frequency content compared to those from the abutments and foundation.
- The 3D numerical models of the Tahtaköprü earthfill, Kavşak Bendi concrete-faced rockfill and Feke II RCC dams can be calibrated using the data and findings in this study, and the earthquake safety of the dams can be determined for different earthquake scenarios that may occur in the future and necessary repair and strengthening measures can be taken.

## Figures and Tables

**Table 1:** Main characteristics of the damaged dams during the 6 February 2023 Kahramanmaraş earthquakes [2,26,30].

Name	Location	Type *	Constr. Year	Crest Length (m)	Height from Foundation (m)	Damage Level
Aslantaş	Osmaniye	ZED	1984	566.0	95.0	Minor cracks along the crest
Kalecik	Osmaniye	ECRD	1985	194.6	80.0	Moderate cracks along the crest
Yarseli	Hatay	ECSGD	1989	960.0	43.5	Moderate cracks along the crest
Bahçe Arıklıkaş	Osmaniye	ED	2000	355.0	25.0	Major cracks along the crest
Samandağ Karamanlı	Hatay	ED	2005	406.0	26.0	Minor cracks along the crest
Hassa Demrek	Hatay	ED	2006	503.0	26.0	Minor cracks along the crest
Büyük Karaçay	Hatay	CFRD	2017	415.1	105.0	Minor cracks along the crest
Kırıkhan Kutlusoğuksu	Hatay	ECRD	2017	419.1	40.0	Major cracks along the crest
Reyhanlı	Hatay	ED	2020	9271.0	28.2	Major cracks along the crest
Sürgü	Malatya	ECRD	1969	736.0	57.0	Moderate cracks along the crest
Sultansuyu	Malatya	ECSGD	1992	721.3	60.0	Major cracks along the crest
Çetintepe	Adıyaman	ECRD	2022	780.0	116.0	Moderate cracks along the crest
Kartalkaya	Kahramanmaraş	ZED	1972	205.0	57.0	Major cracks along the crest
Kılavuzlu	Kahramanmaraş	ECSGD	2014	556.0	61.0	Moderate cracks along the crest
Islahiye Bayraktepe	Gaziantep	ECSGD	2017	233.5	41.0	Minor cracks along the crest
Nurdağı Hamidiye	Gaziantep	ECRD	2018	182.0	29.0	Moderate cracks along the crest
Yukarı Afrin	Kilis	ECSGD	2015	720.2	60.0	Minor cracks along the crest

* ZED: zoned earthfill dam, ECRD: earth core rockfill dam, ECSGD: earth core sand gravel dam, ED: earthfill dam, CFRD: concrete-faced rockfill dam.

**Table 2:** Comparison of the peak ground accelerations and the ratios recorded at dam crest, left and right abutments for the M7.7 mainshock.

Location		Peak Ground Acceleration (cm/s^2^)								
	E-W	N-S	U	Ratio						
				N-S/E-W	U/E-W	U/N-S	Crest/LA		Crest/RA	LA/RA
Crest	228.72	253.85	165.84	1.11	0.73	0.65	E-W	1.68	0.77	0.46
Left Abut. (LA)	135.89	157.01	114.20	1.16	0.84	0.73	N-S	1.62	0.68	0.42
Right Abut. (RA)	298.11	373.87	166.51	1.25	0.56	0.45	U	1.45	0.99	0.69

**Table 3:** Comparison of peak ground accelerations and the ratios recorded at dam crest, left and right abutments for the M6.6 aftershock.

Location		Peak Ground Acceleration (cm/s^2^)								
	E-W	N-S	U	Ratio						
				N-S/E-W	U/E-W	U/N-S	Crest/LA		Crest/RA	LA/RA
Crest	178.82	82.25	72.19	0.46	0.40	0.88	E-W	3.32	2.50	0.75
Left Abut. (LA)	53.83	35.30	23.24	0.66	0.43	0.66	N-S	2.33	1.52	0.65
Right Abut. (RA)	71.41	54.11	38.79	0.76	0.54	0.72	U	3.11	1.86	0.60

**Table 4:** Comparison of the peak ground accelerations recorded at dam crest (TK0140) and right abutment (TK0141) stations for the M7.7 and M7.6 earthquakes.

Location	Peak Ground Acceleration (cm/s^2^)					
	E-W		N-S		U	
	M7.7	M7.6	M7.7	M7.6	M7.7	M7.6
Crest	25.92	86.75	34.22	60.48	18.61	53.29
Right Abut. (RA)	16.61	191.35	12.97	77.99	14.45	74.93
Ratio of Crest/RA	1.56	0.45	2.64	0.78	1.29	0.71

**Table 5:** Comparison of peak ground accelerations recorded at the dam crest (TK0133) and foundation (TK0134) stations for the M7.7 and M7.6 earthquakes.

Location	Peak Ground Acceleration (cm/s^2^)					
	E-W		N-S		U	
	M7.7	M7.6	M7.7	M7.6	M7.7	M7.6
Crest	74.04	79.31	77.18	44.67	39.55	36.32
Foundation	45.20	46.50	68.26	52.74	38.22	35.52
Ratio of Crest/Foundation	1.64	1.71	1.13	0.85	1.03	1.02

## Data Availability

The datasets generated during and/or analyzed during the current study are available from the corresponding author on reasonable request.
